# Secukinumab: Rapid Efficacy in Psoriasis After Primary Failure With Ustekinumab and Adalimumab

**DOI:** 10.5826/dpc.1004a102

**Published:** 2020-10-26

**Authors:** Aline Lissa Okita, Tatiane Benini, Denise Reis Longhi

**Affiliations:** 1 Department of Dermatology, Universidade de Mogi das Cruzes, São Paulo, Brazil

**Keywords:** severe psoriasis, biologics, secukinumab, adalimumab, ustekinumab

## Introduction

Several guidelines recommend phototherapy, systemic agents, or biologic therapy for the treatment of moderate-to-severe psoriasis. Sequential use of more than 1 biologic has become more common due to primary or secondary failure with this type of treatment and an increase in the number of drugs available. However, the order of usage of these drugs is still speculative. We report about a patient with severe psoriasis with primary failure to respond after treatment with ustekinumab and adalimumab who achieved psoriasis area and severity index (PASI) 100 score with secukinumab in 8 weeks.

## Case Presentation

A 47-year-old woman presented with a 30-year history of psoriasis vulgaris without psoriatic arthritis or other comorbidities. She was previously treated with methotrexate and PUVA but had hepatotoxicity and minimal response with UVB narrowband.

**Figure 1:**
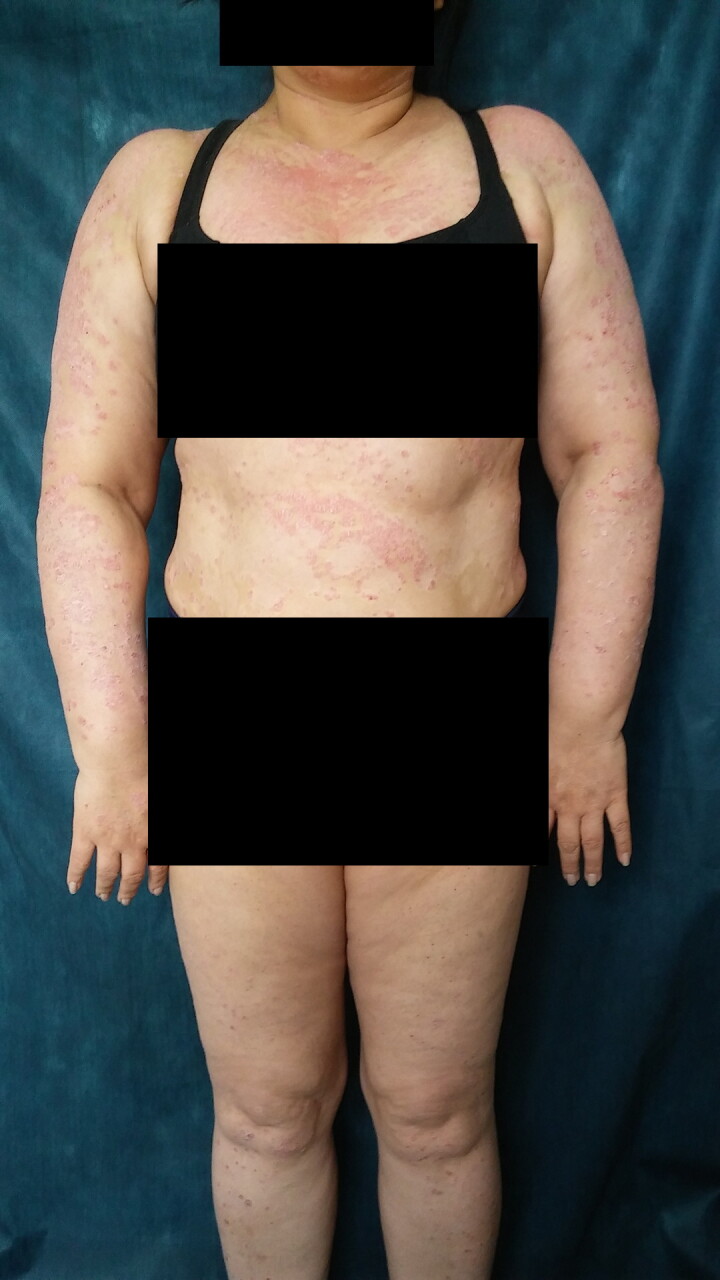
Clinical presentation prior to ustekinumab treatment.

**Figure 2:**
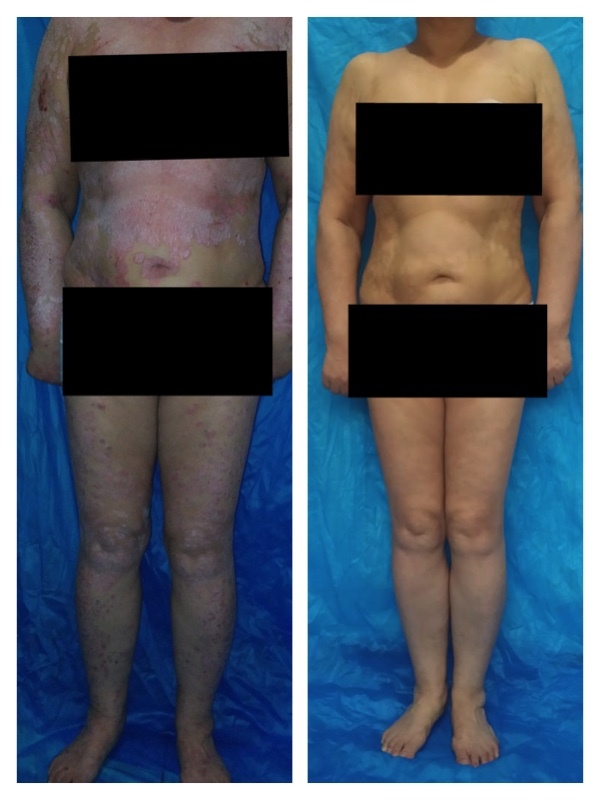
Clinical presentation. (A) After adalimumab treatment. (B) After 5 weeks of secukinumab treatment.

**Figure 3:**
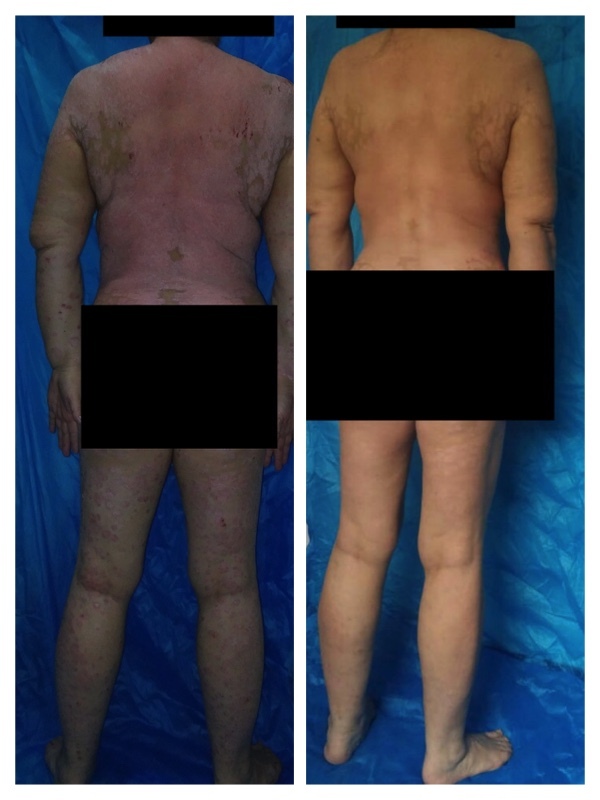
Clinical presentation. (A) After adalimumab treatment. (B) After 5 weeks of secukinumab treatment.

At first visit, the patient revealed PASI 23.7, dermatology life quality index (DLQI) 30, body surface area (BSA) 30, and body weight 80 kg (Figure 1) and was subsequently treated with ustekinumab 45 mg for 6 months with no response. Then she received adalimumab, but symptoms worsened with an increased number of lesions and intense itching (PASI 33.6, DLQI 30, BSA 78) (Figures 2A and 3A). After that, she received secukinumab 300 mg at weeks 0, 1, 2 and 3, 4 achieving PASI 75 at week 5 (Figures 2B and 3B) and PASI 100 and DLQI 0 at week 8.

## Conclusions

Studies on secukinumab have demonstrated rapid and high efficacy in the treatment of psoriasis, and bio-naïve patients achieved higher scores, PASI 90, than those previously exposed to treatment with biologics.

However, many authors reported successful treatment with secukinumab after biological exposure. In one series of 6 patients, patients exhibited efficacy with secukinumab after failure with ustekinumab. Four patients had primary failure with ustekinumab and 2 patients experienced secondary failure. After 12 weeks, 4 patients achieved PASI 90 with secukinumab [1].

Another study of 235 patients randomized 3 groups of anti-TNF nonresponders to treatment with secukinumab at 300 or 150 mg. The 3 groups were: patients with primary failure to anti-TNFα; patients with secondary failure to anti-TNFα; and patients who failed with more than one anti-TNFα. The patients in the 3 groups achieved statistically significant rates of PASI 75 using secukinumab 300 mg [2].

Magnano et al reported on 16 psoriatic patients previously treated with more than 1 systemic or biologic agent, without control of skin lesions. These patients were treated with secukinumab, and 8 of them obtained a complete clearance (PASI 100), 6 patients presented PASI 90, and 2 patients showed PASI 75. All patients presented improvement in the DLQI [3]. In addition, a case of successful treatment with secukinumab in recalcitrant psoriatic arthritis treated previously with 2 anti-TNF drugs was reported [4].

The case reported here was exposed to multiple systemic treatments resulting in inadequate disease control and adverse events that led to discontinuation of therapies. She failed to respond to both anti-TNFα and anti-IL12/23 drugs but reached PASI 100 in 8 weeks with secukinumab without any adverse effects. This indicates that each patient may have a specific behavior in the disease pathway and might respond better to certain drugs. Further studies are needed to determine which factors are essential to define the biologic of choice.
